# A Novel Real-Time SYBR Green PCR Assay for Integron Profiling in *Pseudomonas aeruginosa*: Unveiling the Resistance Nexus Between Food and Clinical Isolates

**DOI:** 10.3390/pathogens15080788

**Published:** 2026-07-24

**Authors:** Hend Emad, Mahmoud M. Amer, Munirah M. Alhammadi, Mona Alharbi, Yasmen F. Mahran, Attia A. Attia, Khalid Maniah, Azza SalahEldin El-Demerdash

**Affiliations:** 1 Department of Botany and Microbiology, Faculty of Science, Benha University, Benha 13518, Egypt; 2 Department of Biology, College of Science, Princess Nourah bint Abdulrahman University, P.O. Box 84428, Riyadh 11671, Saudi Arabia; mmalhammadi@pnu.edu.sa (M.M.A.); mawalharbi@pnu.edu.sa (M.A.); 3 Department of Pharmacology and Toxicology, Faculty of Pharmacy, Ain Shams University, Cairo 11566, Egypt; jassie_81@hotmail.com; 4 Department of Biology, King Khalid Military Academy, P.O. Box 22140, Riyadh 11495, Saudi Arabia; khalede_sa@hotmail.com; 5 Department of Biotechnology, Agricultural Research Center (ARC), Animal Health Research Institute (AHRI), Zagazig 44516, Egypt

**Keywords:** epidemiology, One Health, resistome, pandrug, plasmid-borne integrons, Pseudomonas

## Abstract

**Background:** The dissemination of extensively drug-resistant *Pseudomonas aeruginosa* strains across the human–food interface represents a critical threat to global public health, complicating empirical therapeutic choices and challenging traditional nosocomial restriction paradigms. **Objective:** This study investigated the prevalence, phenotypic resistome landscapes, and underlying integron-mediated mobilization mechanics of *P. aeruginosa* across the One Health continuum using an optimized real-time quantitative PCR (qPCR) platform. **Methods:** A total of 116 samples comprising human clinical specimens (*n* = 61) and retail food matrices (*n* = 55) were screened. Phenotypic resistance was profiled against 16 antipseudomonal agents, with polymyxin and colistin resistance validated via reference broth microdilution. A novel real-time SYBR Green qPCR assay backed by derivative melting curve analysis was developed to track Class 1 (*intI1*) and Class 2 (*intI2*) integrons. **Results:** *Pseudomonas aeruginosa* was recovered from 36.2% (*n* = 42/116) of total samples, exhibiting a homogeneous distribution across clinical (32.8%) and retail food matrices (40.0%, *p* = 0.445). Antibiogram characterization revealed a critical resistance burden: 100% of isolates fell into extreme epidemiological tiers, with 71.4% (*n* = 30) exhibiting an extensively drug-resistant (XDR) phenotype and 28.6% (*n* = 12) reaching absolute pandrug-resistant (PDR) status. Absolute phenotypic resistance (100%) was recorded across both cohorts for meropenem, ceftazidime, aztreonam, tobramycin, polymyxins, and novel combination therapeutics (ceftazidime–avibactam and ceftolozane–tazobactam). Pairwise tracking demonstrated an exceptionally strong positive correlation in resistance distribution patterns between human and food isolates (Spearman’s rho = 0.948, *p* < 0.001). Analytical validation of the qPCR assay demonstrated tight, reproducible specific derivative melting peaks (T_m_) at 83.2 °C for *intI1* and 83.8 °C for *intI2*. Genotypic screening revealed universal conservation of plasmid-borne *intI1* (100%), whereas plasmidic *intI2* was variably distributed (61.9%). Crucially, *intI2* carriage served as a definitive marker for the transition to complete pandrug resistance, being detected in 100% (*n* = 12/12) of PDR isolates compared to 46.7% (*n* = 14/30) of XDR strains (*p* = 0.001), supported by a strong positive correlation with the PDR phenotype (rho = 0.496, *p* = 0.0008). **Conclusions:** These findings reveal an overlapping, extreme resistome across the food-clinical interface and identify *intI2* as a genetic tipping point for absolute pandrug resistance. The validated real-time assay provides a highly sensitive, proactive molecular surveillance framework capable of mapping high-risk mobile genetic platforms before they manifest as catastrophic empirical treatment failures.

## 1. Introduction

*Pseudomonas aeruginosa* is a highly adaptable, opportunistic pathogen capable of causing severe, life-threatening infections, particularly in immunocompromised individuals and hospitalized patients [1,2]. Renowned for its intrinsic resistance to a wide range of antimicrobial agents, this bacterium possesses an extraordinary capacity to acquire novel resistance mechanisms through horizontal gene transfer (HGT) [3,4]. In recent years, the emergence of multidrug-resistant (MDR), extensively drug-resistant (XDR), and pandrug-resistant (PDR) strains of *P. aeruginosa* has severely compromised global healthcare, rendering conventional empiric therapies ineffective and drastically increasing patient mortality rates [5,6].

While historically viewed primarily as a nosocomial threat, *P. aeruginosa* is increasingly recognized as a critical foodborne contaminant [7]. Its presence in fresh produce, meat, dairy, and water systems highlights a troubling bridge between agricultural environments and clinical settings [8,9]. The food chain serves as a vast, under-monitored reservoir for antibiotic-resistant bacteria, facilitating the dissemination of resistance traits from farm to fork, and ultimately, to human hosts [10,11].

Central to the rapid evolution and spread of this resistance are integrons-site-specific recombination systems capable of capturing, expressing, and rearranging mobile gene cassettes, which frequently encode resistance to critical antimicrobials [12,13]. Among these, Class 1 and 2 integrons are most notably implicated in the dissemination of multidrug resistance traits [14,15].

Determining the precise correlation between the presence of specific integron classes and severe phenotypic resistance profiles (MDR, XDR, and PDR) is paramount to understanding the trajectory of resistance evolution across food and clinical ecosystems.

Traditional endpoint PCR methods for detecting integrons, while useful, often lack the speed, sensitivity, and quantitative precision required for rapid epidemiological surveillance and clinical diagnostics. Real-time PCR (qPCR) assays offer a robust alternative, providing rapid and highly specific detection. However, there remains a critical need for cost-effective, high-throughput, and accessible molecular tools capable of simultaneously profiling diverse integron classes without the prohibitive costs associated with fluorogenic probes.

To address this gap, this study introduces a novel, highly sensitive real-time SYBR Green PCR assay designed for the rapid detection and differentiation of integron classes. Using this newly developed molecular tool, we investigated the prevalence and distribution of integrons in *P. aeruginosa* strains isolated from both clinical patients and various food samples. Furthermore, we evaluated the direct correlation between these genetic elements and the phenotypically manifested MDR, XDR, and PDR patterns. By bridging environmental food monitoring with clinical surveillance, this research aims to provide critical insights into the molecular epidemiology of *P. aeruginosa* and offer an efficient diagnostic asset for global antimicrobial resistance (AMR) stewardship.

## 2. Materials and Methods

During the preparation of this manuscript, the author(s) used Gemini (Google, July 2026 version) for the purposes of assisting in the initial drafting, structuring, and language refinement of the Materials and Methods section.

### 2.1. Sample Collection and Processing

A total of 116 non-repetitive samples were prospectively collected across clinical and food matrices between January 2026 and May 2026.

Human Clinical Specimens (*n* = 61): Clinical specimens were collected from patients admitted to Benha University Hospital (Benha, Qalyubia Governorate, Egypt), comprising blood (*n* = 29), urine (*n* = 21), and burn wound swabs (*n* = 11). Specimen collection was integrated into routine diagnostic workflows. Written informed consent was proactively obtained from all participating patients (or their legal guardians) at the time of specimen collection, ensuring complete anonymity and permission for academic research use. The research protocol and specimen utilization were formally approved by the Research Ethics Committee (REC) of the Faculty of Science, Benha University, Egypt (Approval Code: BUFs-REC-2026-544 Bot; Approval Date: 3 June 2026).

Food Samples (*n* = 55): Concurrently, retail food samples consisting of raw milk (*n* = 25) and minced meat (*n* = 30) were purchased from random local retail markets and butcher shops in Benha, Qalyubia Governorate, Egypt. All food samples were collected in sterile containers, transported to the laboratory under chilled conditions (4 °C), and processed within 2 h of collection.

### 2.2. Isolation and Phenotypic Identification of Pseudomonas aeruginosa

Clinical specimens (blood, urine, and swabs) were immediately inoculated onto MacConkey agar and Blood agar plates (Oxoid, Basingstoke, UK). Concurrently, food matrices were subjected to initial pre-enrichment in Buffered Peptone Water (BPW) before being subcultured onto selective Cetrimide Agar plates. All plates were incubated under aerobic conditions at 37 °C for 24–48 h. Presumptive colonies displaying characteristic pyocyanin pigment production, a grape-like odor, and Gram-negative rod morphology were subjected to standard biochemical verification, including oxidase, catalase, motility, and growth at 42 °C [16]. Following preliminary biochemical screening, phenotypic confirmation of the presumptive *Pseudomonas aeruginosa* isolates was performed at the species level using standardized API 20E test strips (BioMérieux, Marcy-l’Étoile, France) in strict accordance with the manufacturer’s instructions.

### 2.3. Antimicrobial Susceptibility Testing (AST) and Phenotypic Classification

Phenotypic antimicrobial resistance profiles for the confirmed *P. aeruginosa* isolates were determined using the Kirby-Bauer disk diffusion method [17] on Mueller–Hinton agar, according to the Clinical and Laboratory Standards Institute (CLSI) guidelines [18]. The antipseudomonal antibiotic disk panel spanned 8 distinct functional classes:

- **Aminoglycosides:** Amikacin (AK, 30 µg), Gentamicin (CN, 10 µg), Tobramycin (TOB, 10 µg).
- **Carbapenems:** Imipenem (IPM, 10 µg), Meropenem (MEM, 10 µg).
- **Cephalosporins:** Ceftazidime (CAZ, 30 µg), Cefepime (FEP, 30 µg).
- **Fluoroquinolones:** Levofloxacin (LEV, 5 µg), Ciprofloxacin (CIP, 5 µg)
- **Penicillins + beta-lactamase Inhibitors:** Piperacillin–Tazobactam (TZP, 100/10 µg), Ticarcillin–Clavulanic acid (TIM, 75/10 µg),
- **Monobactams:** Aztreonam (ATM, 30 µg).
- **Polymyxins:** Polymyxin B (PB, 300 units), Colistin (CT, 10 µg) and susceptibility determined exclusively via broth microdilution; MIC range: 0.25 to 1024 µg [19,20].
- **Beta-lactam\beta-lactamase Inhibitor Combinations:** Ceftazidime–Avibactam (CZA, 30/20 µg), Ceftolozane–Tazobactam (C/T, 30/10 µg).

The Multiple Antibiotic Resistance (MAR) index was determined for each isolate using the formula proposed by Tambekar et al. [21]. An MAR index greater than the critical threshold of 0.20 identified isolates from high-risk sources under significant antibiotic selective pressure, linking clinical exposure and agricultural practices to the evolution of both the foodborne and clinical isolates. Additionally, isolates were classified into resistance tiers according to international consensus definitions [22] incorporating the precise MIC values obtained for the polymyxin class: Multidrug-Resistant (MDR) defined as non-susceptibility to ≥1 agent in ≥3 antimicrobial categories; Extensively Drug-Resistant (XDR) defined as non-susceptibility to ≥1 agent in all but ≤2 categories; and Pandrug-Resistant (PDR) defined as non-susceptibility to all tested agents across all antimicrobial categories.

### 2.4. Plasmid Extraction

Plasmid DNA was extracted using the GeneJET Plasmid Miniprep Kit (Thermo Scientific, Schwerte, Germany) according to the manufacturer’s instructions. To bias the detection toward mobile extrachromosomal elements, an alkaline-lysis-based extraction protocol was selectively employed to yield a highly plasmid-enriched template. Neutralization and centrifugation steps were strictly timed to maximize genomic DNA precipitation, thereby minimizing chromosomal DNA carryover. While trace chromosomal background cannot be entirely eliminated by spin-column chromatography, the resulting high-purity plasmid-enriched eluates were utilized as the primary templates to track plasmid-borne integron classes, which are of primary epidemiological concern for horizontal gene transfer. The concentration and purity of the isolated DNA templates were determined spectrophotometrically (A_260_/A_280_ ratio), and the extracts were stored at −20 °C until downstream real-time PCR amplification.

### 2.5. Real-Time SYBR Green qPCR Optimization and Analytical Validation for Integron Classes 1 and 2

To ensure high-throughput epidemiological tracking, a real-time quantitative PCR (qPCR) assay utilizing SYBR Green chemistry was optimized for the specific detection and profiling of plasmidic Class 1 (*intI1*) and Class 2 (*intI2*) integron genes. The oligonucleotide primers utilized in this study (detailed in Table 1) were structurally designed and synthesized de novo using the Primer3 (v. 4.1.0) and FastPCR software (version 6.7, PrimerDigital, Helsinki, Finland). These sequences were systematically validated in silico to ensure maximum target conservation across diverse strains, optimize thermodynamic compatibility, and eliminate potential primer-dimer configurations or multi-locus cross-reactivity.

To ensure strict analytical validation and monitor the accuracy of the real-time assay, well-characterized positive control plasmids from previously established lineages were utilized [12]. Specifically, a confirmed plasmid template extracted from *Salmonella* Birkenhead (strain MAS2016; GenBank accession number: KY009928) served as the positive validation control for the Class 1 integron (*intI1*) assay. Concurrently, a plasmid template from *Escherichia coli* O158 (strain MAS5/Egy/16; GenBank accession number: KY070366) was employed as the positive validation control for the Class 2 integron (*intI2*) assay. These positive controls were processed under identical extraction and amplification parameters alongside the test isolates.

To determine the analytical sensitivity, linearity, and amplification efficiency of the assay, standard curves were constructed for both *intI1* and *intI2* targets using 10-fold serial dilutions of the control plasmids ranging from 10^8^ to 10^1^ copies/µL. The assay demonstrated high linearity, yielding correlation coefficients (R^2^) of 0.998 for *intI1* and 0.996 for *intI2*, with corresponding amplification efficiencies of 98.5% (slope: −3.36) and 96.2% (slope: −3.42). The empirical Limit of Detection (LOD) for both targets was determined to be 10 copies per reaction. Assay precision and reproducibility were evaluated by measuring intra-run and inter-run precision across three concentration tiers (10^6^, 10^4^, and 10^2^ copies/µL) in triplicate. The coefficients of variation (CV%) for the quantification cycle (C_t_) values did not exceed 1.8% for intra-run and 2.3% for inter-run evaluations.

The real-time molecular assay was executed in a 20 µL reaction matrix containing 10 µL of 2× HERA SYBR^®^ Green RT-qPCR Master Mix (Willowfort, Nottingham, UK), 0.5 µM of each forward and reverse primer (0.4 µM final concentration), 4 µL of nuclease-free water, and 5 µL of the purified plasmid DNA template.

Amplification kinetics were driven by an initial enzymatic activation and denaturation at 95 °C for 5 min, followed by 40 cycles of denaturation at 95 °C for 15 s, and a unified annealing/extension step at 60 °C for 60 s. A conservative C_t_ cutoff threshold was established at 35 cycles.

A no-template control (NTC), substituting the DNA template with nuclease-free water, was systematically included in every operational run to monitor for reagent contamination and primer-dimer artifacts. Immediately following the final amplification cycle, a post-amplification continuous melting curve analysis was executed to verify product specificity. The temperature profile involved an initial equilibration hold at 60 °C for 1 min, followed by a gradual temperature increase from 60 °C to 95 °C at a continuous ramp rate of 0.5 °C with continuous fluorescence acquisition, concluding with a final hold at 95 °C for 15 s. The resulting amplification profiles and derivative melting peaks (−dF/dT) were analyzed using the thermocycler system software.

### 2.6. Statistical Analysis

Data compilation, management, and descriptive statistics were performed using R software version 4.3.3 (R Project for Statistical Computing, https://www.r-project.org/) and IBM SPSS Statistics version 26 (IBM Corp., Armonk, NY, USA). The prevalence of *Pseudomonas aeruginosa* among different sample sources (food-clinical axis) and the distribution of antimicrobial resistance patterns and integron genes among isolates from various origins were evaluated using Pearson’s chi-square test or Fisher’s Exact Test when expected cell frequencies were less than 5. Differences in integron distribution between food and clinical isolates were assessed using contingency table analyses. The Class 1 integron gene (*intI1*), TOB, MEM, CAZ, ATM, PB, CZA, and C/T phenotypes were excluded from association and correlation analyses because they were detected in all investigated isolates and therefore lacked variability. Associations among Class 2 integron carriage (*intI2*), antimicrobial resistance traits, and resistance phenotypes (XDR and PDR) were further explored using correlation analysis and visualized as a correlation heatmap in R software (v4.3.3.). The similarity between human and food *Pseudomonas aeruginosa* isolates with respect to antimicrobial resistance phenotypes, resistance categories, and integron gene prevalence was explored using Spearman rank correlation analysis based on the percentage occurrence of each characteristic. Pairwise relationships were visualized using the ggpairs function of the GGally package in R software (v4.3.3.). Statistical significance thresholds were strictly set at a two-tailed *p*-value < 0.05.

## 3. Results

### 3.1. Prevalence and Distribution of Pseudomonas aeruginosa

Out of a total of 116 examined samples across the clinical and food ecosystems, *Pseudomonas aeruginosa* was successfully isolated from 42 samples, establishing an overall prevalence rate of 36.2% (Table 2). Within the human clinical cohort (*n* = 61), the pathogen exhibited an isolation rate of 32.8% (*n* = 20). Among the clinical matrices, the highest prevalence was observed in burn wound swabs at 36.4% (*n* = 4/11), followed closely by blood cultures at 34.5% (*n* = 10/29) and urine specimens at 28.6% (*n* = 6/21).

In comparison, the food reservoir (*n* = 55) demonstrated a higher overall isolation rate of 40.0% (*n* = 22). Interestingly, this recovery rate was perfectly uniform across both tested food matrices, with both minced meat (*n* = 12/30) and raw milk (*n* = 10/25) yielding a prevalence of exactly 40% (Table 2).

To determine if the distribution of *P. aeruginosa* was biased toward a specific origin, categorical probability tests were performed. Statistical analysis revealed that there were no significant variations in the prevalence of *P. aeruginosa* between the broad human and food sources (*p* = 0.445) or across the five distinct individual sample matrices (*p* = 0.925). This statistical homogeneity indicates that *P. aeruginosa* is widely and evenly distributed across both clinical environments and the retail food chain.

### 3.2. Antibiogram and Phenotypic Resistance Profiling

The 42 verified *P. aeruginosa* isolates were screened against a robust panel of 16 antimicrobial agents representing 8 distinct functional classes (Table 3). The phenotypic profiles revealed a critical, near-universal resistance footprint across the entire collection. Complete, absolute phenotypic resistance (100%, *n* = 42) was recorded for seven key therapeutic choices: Tobramycin (TOB), Meropenem (MEM), Ceftazidime (CAZ), Aztreonam (ATM), Polymyxin B (PB), Ceftazidime–Avibactam (CZA), and Ceftolozane–Tazobactam (C/T). Furthermore, exceptionally high resistance rates were observed for Gentamicin (CN; 95.2%, *n* = 40), Colistin (CT; 81%, *n* = 34), and Levofloxacin (LEV; 81.0%, *n* = 34).

Notably, to address the poor diffusion of lipopeptides in agar and secure clinical validation, the susceptibility profiles of all isolates toward the polymyxin class were quantitatively confirmed via the reference broth microdilution (BMD) method. The MIC determinations perfectly mirrored and confirmed the initial disk-based screening profiles. All isolates deemed resistant to Polymyxin B (100%, *n* = 42) and Colistin (81%, *n* = 34) exhibited extremely high Minimum Inhibitory Concentrations (MICs) of ≥64 μg/mL (Supplementary Table S1). These high-level MIC values far exceed the established CLSI clinical resistance breakpoints (≥4 μg/mL), definitively validating the phenotypic classification of these isolates within the extensively drug-resistant (XDR) and pandrug-resistant (PDR) cohorts.

When evaluating the remaining variable agents, resistance rates remained high: Ticarcillin–Clavulanic acid (TIM) was ineffective against 76.2% (*n* = 32) of strains, Ciprofloxacin (CIP) against 71.4% (*n* = 30), Piperacillin–Tazobactam (TZP) against 66.7% (*n* = 28), while Amikacin (AK) and Cefepime (FEP) both exhibited resistance rates of 57.1% (*n* = 24).

A comparative analysis was performed to identify potential differences between food-derived (*n* = 22) and human-derived (*n* = 20) resistomes (Table 3). The resistance rates across both groups track very closely. For instance, Piperacillin–Tazobactam (TZP) resistance was 54.5% in food isolates versus 80% in human isolates (*p* = 0.108), and Imipenem (IPM) resistance was 63.6% in food isolates versus 60% in human strains (*p* = 1.000). Chi-square and Fisher’s exact tests confirmed that no statistically significant variations (*p* > 0.05) exist for any of the tested drugs between the two sources (Table 3). This parallel resistance structure indicates a shared phenotypic resistome across the food-clinical interface.

### 3.3. Multiple Antibiotic Resistance (MAR) Indices and Acquired Resistance Tiers

**Figure 1:**
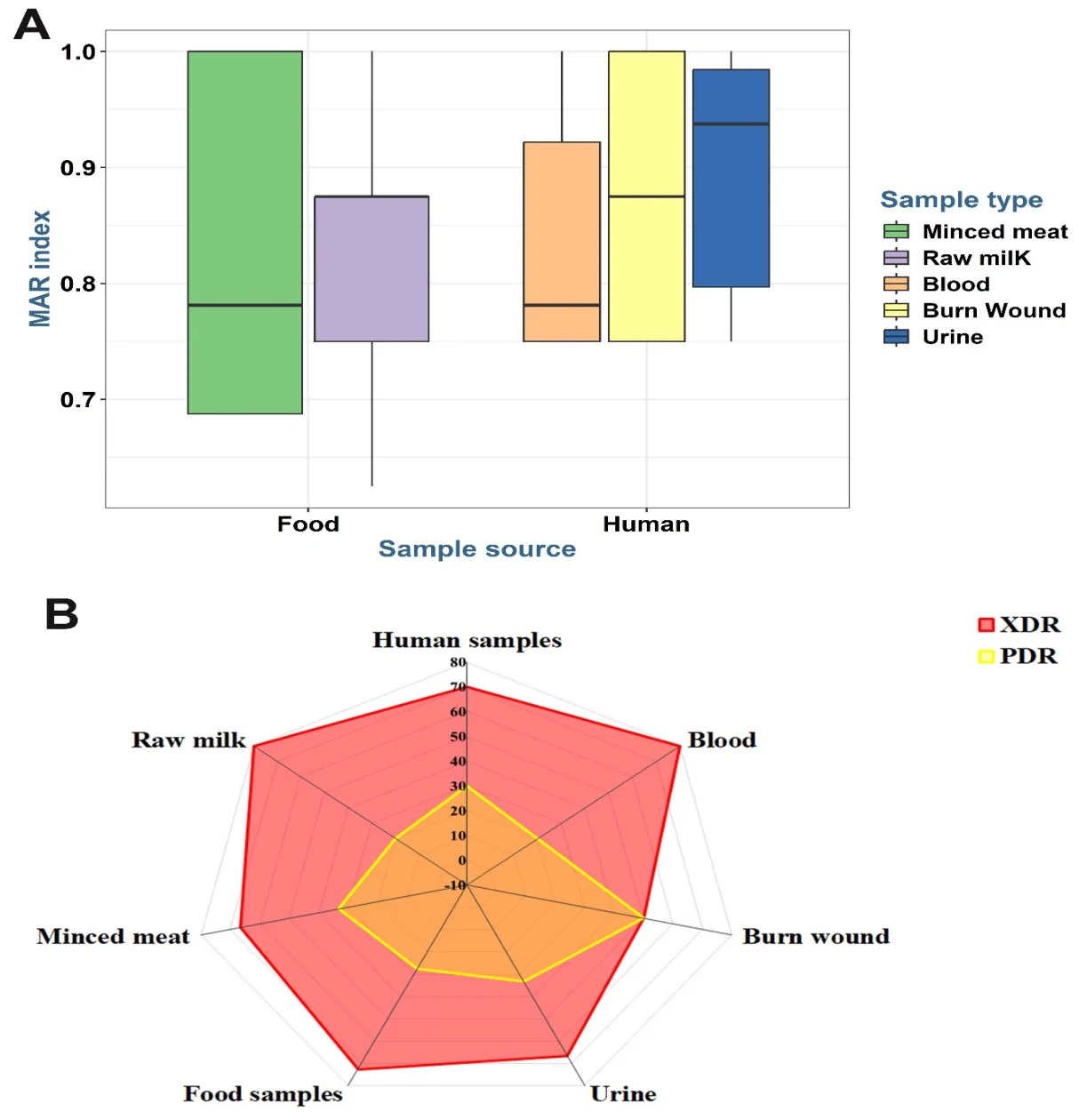
The MAR index (**A**) and frequency of resistance category (**B**) in the tested *Pseudomonas aeruginosa* isolates belonging to various sources.

Meticulous calculation of the Multiple Antibiotic Resistance (MAR) index for each individual isolate revealed a severe baseline risk environment (Figure 1A). Every single one of the 42 confirmed isolates possessed a MAR index ≥ 0.63, vastly exceeding the standard 0.20 threshold that indicates high-risk contamination (Table 4). Strains displaying a MAR index of 0.75 were the most frequent (30.9%, *n* = 13), followed by isolates with an absolute MAR index of 1.00 (28.6%, *n* = 12), which denotes phenotypic resistance to all 16 evaluated antimicrobial agents (Table 4).

Categorization of these profiles into international epidemiological tiers showed that 100% of the isolated population fell into extreme resistance brackets (Figure 1B). Extensively Drug-Resistant (XDR) strains defined here by resistance to 10 to 15 individual agents spanning 7 classes comprised 71.4% (*n* = 30) of the total isolates (Table 4 and Table 5). The remaining 28.6% (*n* = 12) achieved Pandrug-Resistant (PDR) status, showing non-susceptibility to every single agent across all 8 classes (Supplementary Table S2).

Proportional testing showed no significant differences in the distribution of MAR scores or resistance tiers between settings (*p* > 0.05). XDR frequencies were nearly identical between food (72.7%, *n* = 16/22) and human sources (70.0%, *n* = 14/20; *p* = 1.000). Similarly, PDR frequencies showed no significant variation, appearing in 27.3% (*n* = 6/22) of food isolates and 30.0% (*n* = 6/20) of human clinical isolates (Supplementary Table S2).

### 3.4. Molecular Detection of Integron Genotypes and Assay Validation

**Figure 2:**
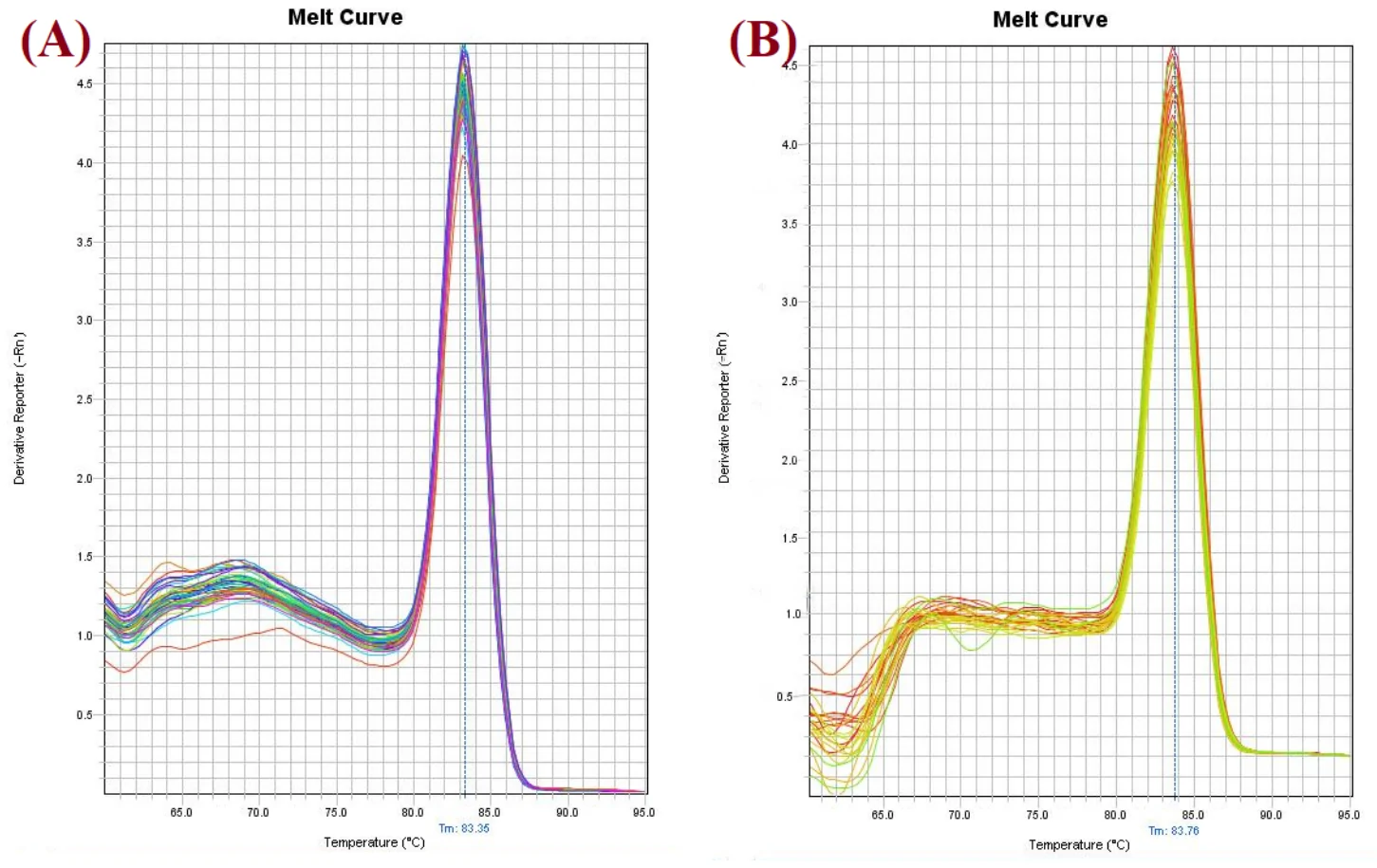
Derivative melting curve profiles (−dF/dT versus Temperature) validating the analytical specificity of the optimized real-time SYBR Green qPCR assays. (**A**) Representative target amplicon curve for Class 1 (*intI1*) showing a distinct symmetric peak centered at 83.35 °C. (**B**) Multi-sample validation overlay profile for Class 2 (*intI2*) demonstrating highly reproducible, tight amplicon melting signatures clustered at 83.76 °C. Universal low-temperature baselines represent typical early-stage dissociation dynamics from plasmid-enriched templates that do not interfere with primary peak resolution.

The diagnostic specificity, structural uniformity, and reproducibility of the optimized real-time qPCR configurations were verified via post-amplification continuous melting curve analysis of the plasmid-enriched templates across all positive *Pseudomonas aeruginosa* isolates (*n* = 42; Figure 2). Amplification of the target sequences yielded prominent, symmetric, and highly reproducible derivative melting peaks (−dF/dT) centered at an average melting temperature (T_m_) of 83.2 °C ± 0.3 °C for Class 1 (*intI1*) amplicons (Figure 2A) and 83.8 °C ± 0.3 °C for Class 2 (*intI2*) amplicons (Figure 2B; representative profiles shown).

A uniform, elevated baseline profile observed across samples in the lower temperature range (65.0 °C–78.0 °C) reflects expected non-specific dye dissociation dynamics typical of dense, plasmid-enriched templates. Crucially, this background fluorescence signal remained entirely resolved and separated from the primary amplicon signals, ensuring that baseline anomalies did not compromise the definitive qualitative scoring or diagnostic accuracy of either target gene. No specific amplification or melting peaks were observed in any of the parallel no-template controls (NTCs), validating the absolute absence of primer-dimer artifacts or external contamination within the reaction matrices.

Real-time SYBR Green PCR amplification was utilized to profile the underlying mobile genetic elements responsible for capturing these resistance traits across the synchronized cohort. The Class 1 integron gene (*intI1*) was ubiquitously conserved, appearing in 100% (*n* = 42) of the isolated *P. aeruginosa* strains (Supplementary Table S3). This absolute prevalence identifies *intI1* as a core genetic vehicle driving multi-drug resistance within this bacterial population across both interfaces.

In contrast, the Class 2 integron gene (*intI2*) exhibited a variable distribution, tracking in 61.9% (*n* = 26/42) of total strains (Supplementary Table S2). Stratification by specimen origin source revealed that *intI2* carriage was proportionally higher among human clinical isolates (70%, *n* = 14/20) than among retail food isolates (54.5%, *n* = 12/22). However, this distribution difference was not statistically significant (*p* = 0.354), indicating that Class 2 integrons are widely distributed across both foodborne and clinical reservoirs, establishing a shared molecular footprint across the One Health interface.

### 3.5. Multi-Variant Clustering and Correlation Analysis

**Figure 3:**
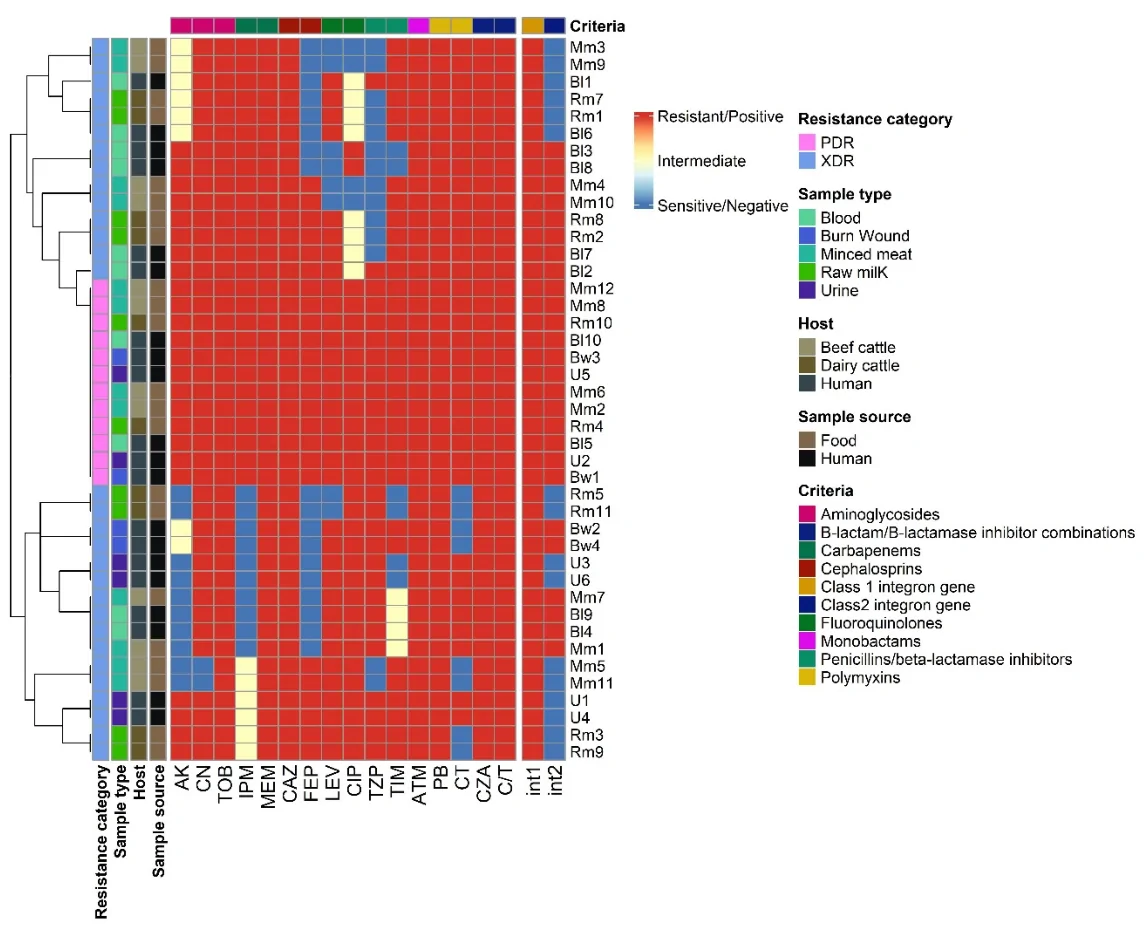
Hierarchical clustering heatmap showing the overall distribution of the investigated *Pseudomonas aeruginosa* isolates based on the phenotypic antimicrobial resistance pattern, and class 1 and 2 integron genes. Different host, sample sources, and types, resistance categories, and antimicrobial classes are color-coded on the right of the heatmap.

**Figure 4:**
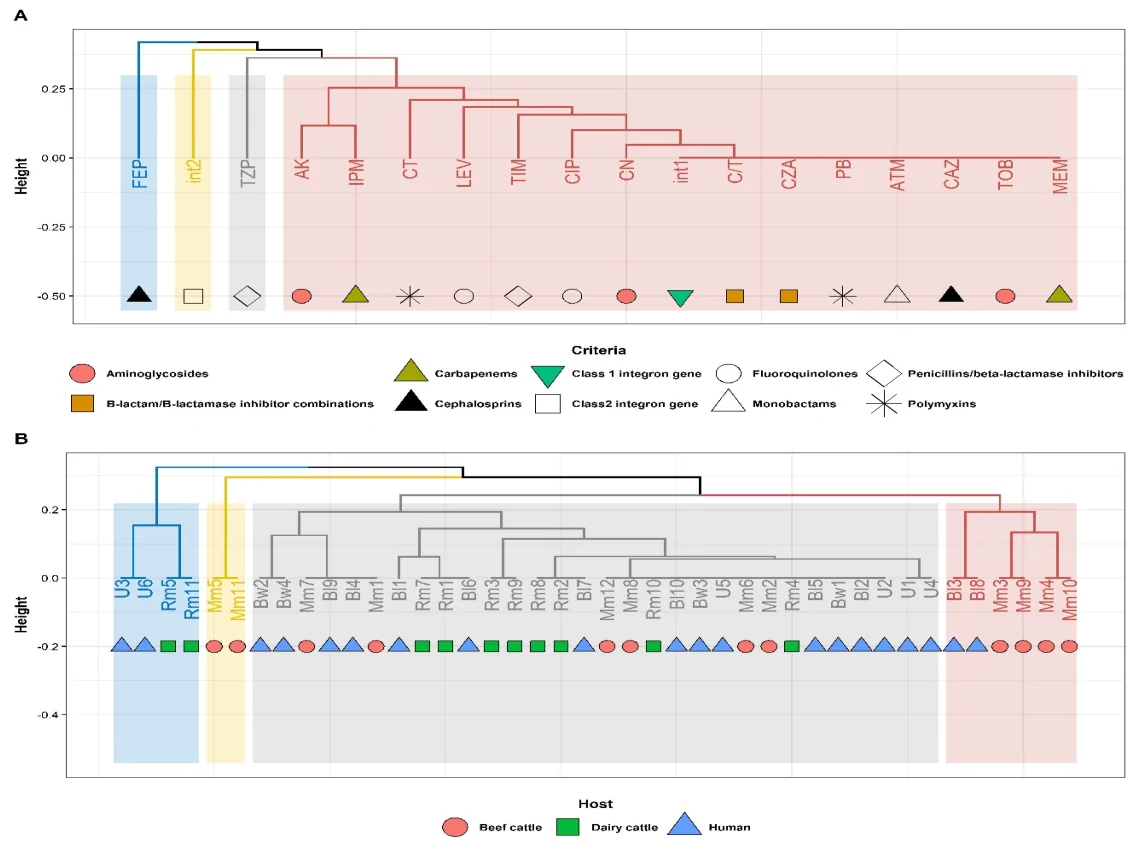
Hierarchical clustering dendrogram displaying the relatedness of the investigated variables (**A**), *Pseudomonas aeruginosa* isolates (**B**), as determined by the phenotypic antimicrobial resistance, and integron genes.

To evaluate the relationships across host types, matrices, phenotypic resistance profiles, and integron genes, multi-variant hierarchical clustering and correlation analyses were performed (Figure 3 and Figure 4). Because *intI1*, TOB, MEM, CAZ, ATM, PB, CZA, and C/T were detected in 100% of the isolates, they lacked statistical variance and were excluded from downstream correlation matrices to avoid mathematical artifacting.

The variable attributes clustered into 4 distinct branches (Figure 4A), highlighting a strong association within a core cluster containing *intI1* along with absolute resistance to TOB, MEM, CAZ, ATM, PB, CZA, and C/T. The isolates themselves exhibited low diversity, grouping into 4 main branches across 13 highly related sub-clusters (Figure 4B). This pattern confirms a close relationship between human and food isolates (Figure 3). This observation is supported by a pairwise scatter plot matrix (Figure 4), which demonstrates a strong, positive correlation in the prevalence of resistance traits and integron elements between human- and food-derived isolates (Spearman’s rho = 0.948, *p* < 0.001).

**Figure 5:**
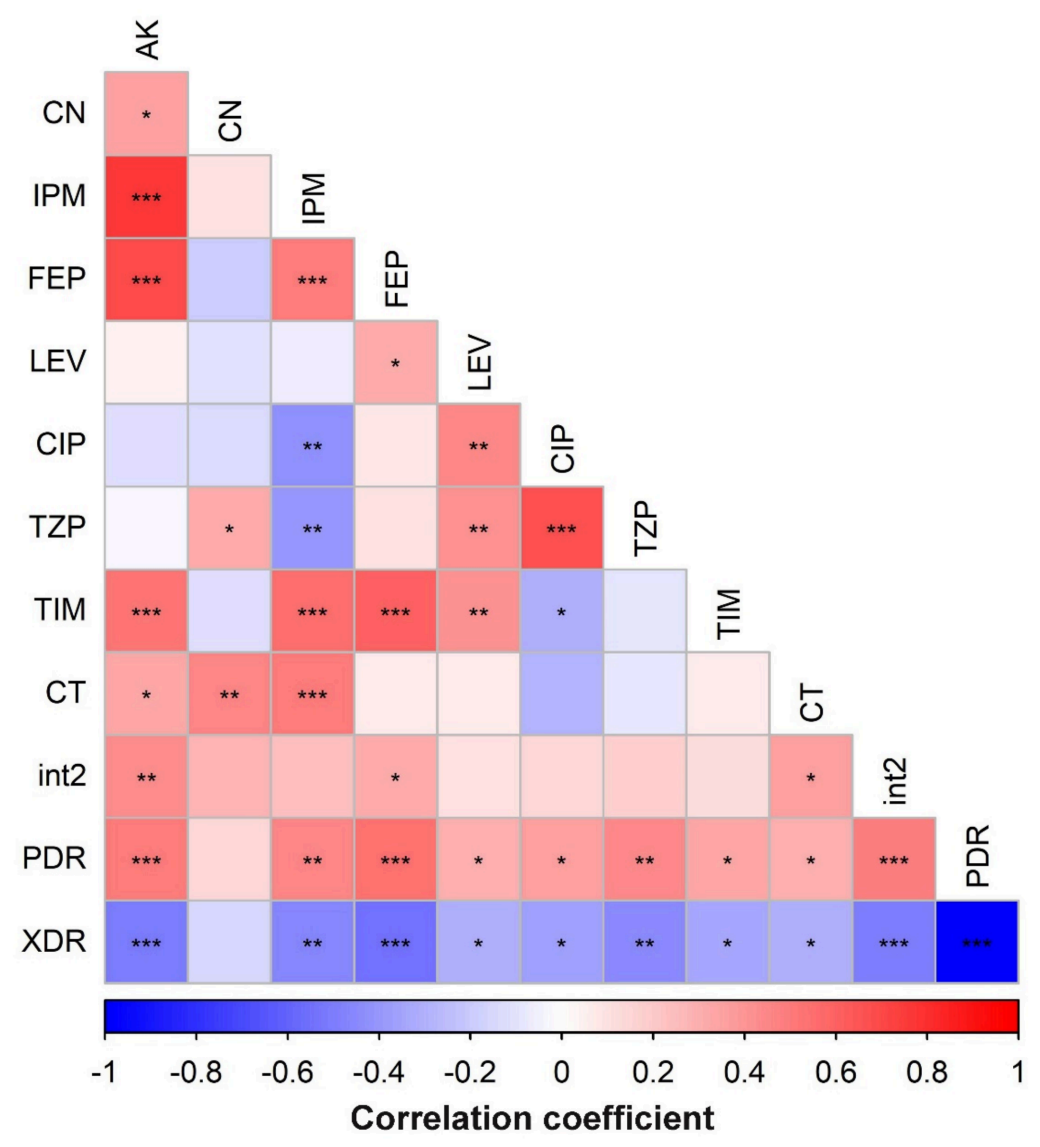
Correlation plot showing pairwise correlations (r) among phenotypic antimicrobial resistance profiles, resistance categories (XDR and PDR), and the Class 2 integron gene (*intI2*) in *Pseudomonas aeruginosa* isolates recovered from human and food sources. The color scale represents the correlation coefficient, with increasing color intensity indicating stronger positive or negative correlations. Asterisks indicate statistically significant correlations (* *p* < 0.05, ** *p* < 0.01, *** *p* < 0.001). Detailed correlation coefficients are presented in Supplementary Figure S1. The Class 1 integron gene (*intI1*), as well as resistance to TOB, MEM, CAZ, ATM, PB, CZA, and C/T, were excluded from association and correlation analyses because they were detected in all investigated isolates and therefore lacked variability.

Pairwise Spearman rank correlation testing (Figure 5 and Supplementary Figure S1) identified significant positive correlations between *intI2* carriage and specific individual drug resistance phenotypes. Specifically, *intI2* presence was significantly correlated with resistance to Amikacin (rho = 0.43, *p* < 0.01), Cefepime (rho = 0.31, *p* < 0.05), and Colistin (rho = 0.37, *p* < 0.05).

Crucially, the structural carriage of the Class 2 integron gene (*intI2*) differed significantly between the XDR and PDR phenotypes (*p* = 0.001, Fisher’s Exact Test; Supplementary Table S4). While *intI2* was distributed in 46.7% (*n* = 14/30) of XDR strains, it was detected in 100% (*n* = 12/12) of PDR strains. This clear link is supported by Spearman coefficients (Figure 5), which show that *intI2* carriage is significantly positively correlated with the progression to a PDR status (rho = 0.496, *p* = 0.0008) and significantly negatively correlated with the XDR tier (rho = −0.496, *p* = 0.0008). These findings indicate that the presence of *intI2* is strongly associated with the accumulation of resistance traits leading to the PDR phenotype.

**Figure 6:**
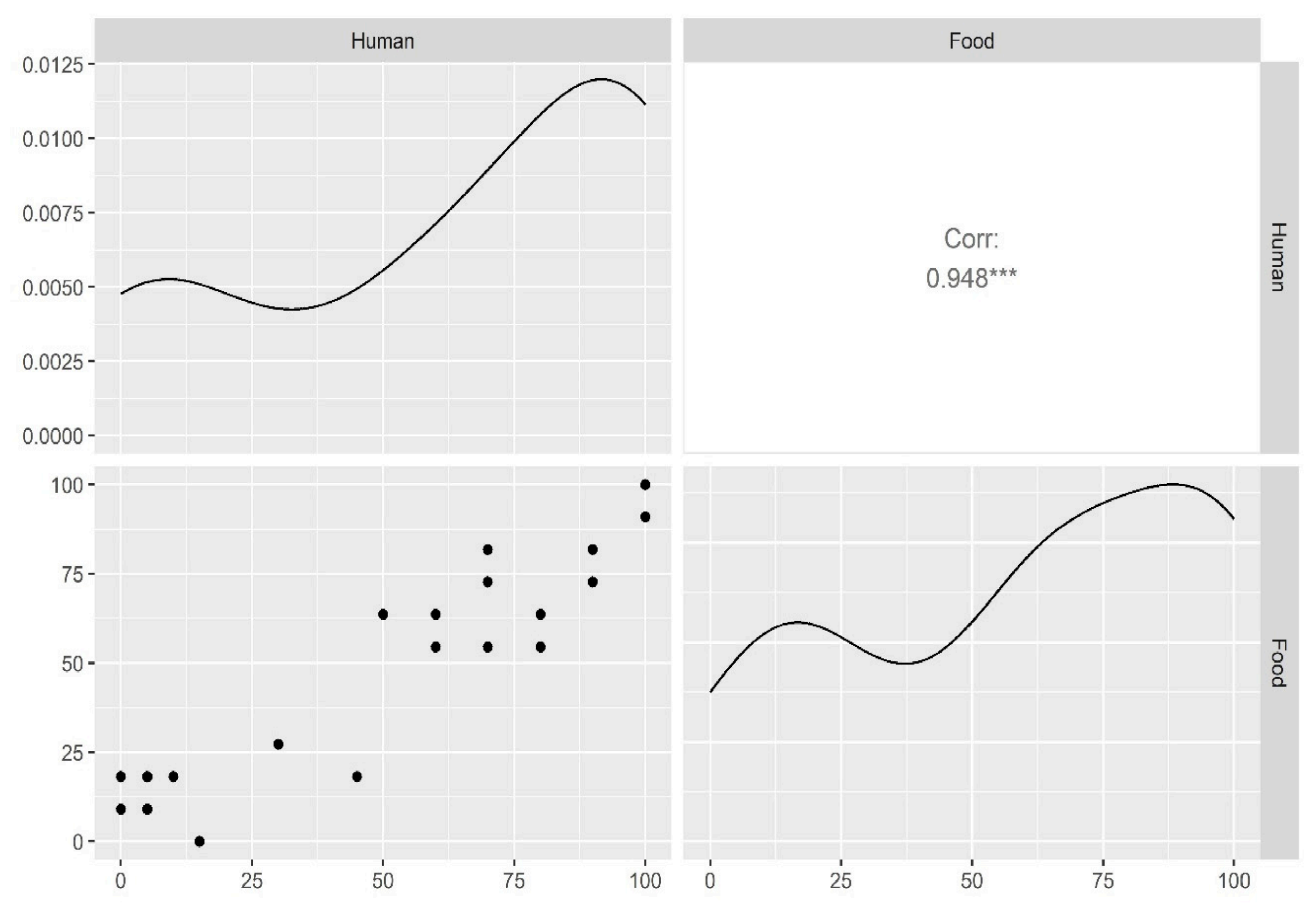
Pairwise scatter plot matrix (pairs plot) showing the correlation between human- and food-derived *Pseudomonas aeruginosa* isolates based on the frequency of antimicrobial resistance phenotypes, resistance categories (XDR and PDR), and integron genes. Spearman’s correlation coefficient (ρ) and significance level are displayed within the plot. *** *p* < 0.001.

Figure 6 illustrates the relationship between human- and food-derived *Pseudomonas aeruginosa* isolates based on the prevalence of antimicrobial resistance phenotypes, resistance categories, and integron genes. Pairwise scatter plot matrix analysis revealed a strong positive correlation between the prevalence patterns observed in human and food isolates (Spearman’s ρ = 0.948, *p* < 0.001). This finding indicates a high degree of similarity in the distribution of antimicrobial resistance traits, resistance categories, and integron genes between isolates originating from the two sources.

## 4. Discussion

The remarkable phenotypic and genotypic homogeneity observed across the human–animal–environmental interface (Spearman’s rho = 0.948, *p* < 0.001) points to a highly correlated phenotypic resistance footprint across these distinct compartments. Rather than functioning as distinct environmental and clinical populations, the strains recovered from retail food matrices (minced meat and raw milk) and human clinical cohorts display strongly overlapping susceptibility patterns. This trend is likely supported by the known ecological versatility and genomic architecture of *P. aeruginosa* [23]. Possessing a large genome (≈5.5–7 Mbp) rich in regulatory genes, two-component systems (such as *phoPQ* and *pmrAB*), and complex transcriptional networks, this pathogen exhibits an extraordinary capacity to adapt to diverse selective pressures [24,25]. When identical classes of antimicrobial agents such as beta-lactams, fluoroquinolones, and aminoglycosides are deployed concurrently in both veterinary medicine and human clinical therapies, they may exert parallel selective pressures [26,27,28]. This directional selection can homogenize resistance phenotypes across separate ecosystems, suggesting that the agricultural food chain may serve as a reservoir for resistance patterns that parallel those observed in intensive nosocomial settings [29,30].

This cross-ecosystem selective pressure is clearly illustrated by the absolute resistance (100%) to the novel, last-resort combinations ceftazidime–avibactam (CZA) and ceftolozane–tazobactam (C/T) among foodborne isolates. Because these advanced therapeutics are restricted exclusively to human clinical settings, their complete failure in food matrices cannot be attributed to direct agricultural exposure [31,32]. Instead, it is hypothesized that this phenotypic profile is maintained by indirect co-selection and cross-resistance mechanisms. In literature, resistance to CZA and C/T typically arises through the overproduction of the chromosomal AmpC beta-lactamase combined with structural mutations within its Omega-loop, or via the horizontal acquisition of Class B metallo-beta-lactamases (such as VIM, IMP, or NDM enzymes) [33]. These metallo-enzymes are structurally capable of degrading advanced cephalosporins while remaining completely uninhibited by avibactam or tazobactam [34].

Crucially, the resistance profiles of these isolates extend beyond β-lactam. Absolute resistance to CZA and C/T was accompanied by extremely high-level resistance to the lipopeptide class, with 100% of Polymyxin B and 81% of Colistin-resistant isolates exhibiting identical Minimum Inhibitory Concentrations (MICs) of ≥64 μg/mL via the gold-standard broth microdilution (BMD) method. While the lack of genomic sequencing prevents the definitive characterization of the molecular mechanisms driving this co-resistance, the co-occurrence of last-resort β-lactam resistance alongside high-level polymyxin MICs indicates a progression toward pandrug-resistant (PDR) phenotypes. The routine use of older, conventional β-lactams and veterinary colistin formulations in livestock production might provide the baseline selective pressure required to maintain both these enzymatic and membrane-associated resistance traits [35,36]. Under this model, agricultural environments could indirectly select for resistance to critical human therapeutics long before these bacterial strains contaminate clinical settings.

At the molecular level, this accumulation of resistance traits is associated with mobile genetic elements, specifically the cooperative interaction between Class 1 (*intI1*) and Class 2 (*intI2*) integrons [13,37]. The universal conservation of *intI1* (100%) across all 42 isolates establishes a baseline genetic platform. Class 1 integrons are highly efficient at capturing and expressing gene cassettes that encode resistance to foundational drugs, such as aminoglycosides (via acetyltransferases like *aac*) and advanced beta-lactams [15]. In this study, the presence of the Class 2 integron (*intI2*) correlated significantly with the transition from an Extensively Drug-Resistant (XDR) state to a complete Pandrug-Resistant (PDR) status (*p* = 0.001). While *intI2* was present in less than half of the XDR isolates (46.7%), it was universally conserved (100%) among PDR strains. These findings suggest that while Class 1 integrons capture primary resistance determinants, the acquisition of a Class 2 integron—often carried on mobile transposons like Tn7 or plasmids—may introduce an independent, complementary site for gene cassette integration [38], potentially expanding the capacity of the cell to accumulate diverse resistance traits.

This hypothesized dual-integron model is further supported by the significant positive correlations identified between *intI2* carriage and resistance to amikacin (rho = 0.43), cefepime (rho = 0.31), and colistin (rho = 0.37). The link to colistin (CT) resistance is particularly critical from a public health perspective. While polymyxin resistance in *P. aeruginosa* frequently stems from chromosomal alterations that modify the lipid A lipopolysaccharide layer (such as the addition of 4-amino-4-deoxy-L-arabinose via the *arnBCADTEF* operon) [39], its correlation with *intI2* in this study suggests a potential mechanism of physical co-localization. If genes governing outer-membrane modifications or plasmid-mediated colistin resistance determinants (such as *mcr* homologues) are physically linked on the same mobile elements as *intI2*, exposure to non-related antibiotics could co-select for colistin resistance [40]. However, high-resolution sequencing remains necessary to confirm the physical linkage of these loci.

Given these complex phenotypic associations, targeted molecular assays provide a valuable tool for surveillance. Phenotypic antibiograms identify resistance only after empirical treatment has failed, and can be confounded by physiological states such as biofilm formation or viable but non-culturable (VBNC) conditions [41]. The development and validation of our novel real-time SYBR Green PCR assay address this limitation by focusing on proactive molecular surveillance. By designing high-affinity primers that target highly conserved, non-mutagenic catalytic regions within the *intI1* and *intI2* integrase genes, the assay circumvents phenotypic lag. The sharp, single-peak thermodynamic profiles obtained during kinetic melting curve analysis confirm the elimination of primer-dimer interference and non-specific amplification, enabling the identification of these genetic elements in complex templates [42]. While the assay does not sequence the complete resistome, its ability to quickly detect target integrases within hours of sample collection offers a valuable, proactive screening tool for identifying strains carrying high-risk mobile genetic platforms.

## 5. Conclusions

This study demonstrates a critical epidemiological shift in *Pseudomonas aeruginosa*, revealing that retail food matrices (minced meat and raw milk) have caught up to intensive clinical environments in harboring hyper-resistant lineages. The near-perfect alignment between human- and food-derived resistomes (rho = 0.948) underscores a continuous, bidirectional mobilization network across the One Health interface. Furthermore, the phenotypic failure of last-resort therapeutics outside of hospital settings indicates a massive expansion of the *P. aeruginosa* resistome, likely maintained via indirect agricultural co-selection.

Rather than remaining confined to stable chromosomal structures, we demonstrate that Class 1 (*intI1*) and Class 2 (*intI2*) integrons are localized on mobile plasmids, transforming them into volatile vehicles for horizontal gene transfer. Within this mobile framework, plasmid-bound *intI2* acquisition serves as the definitive genetic tipping point driving the transition of XDR strains into a pandrug-resistant (PDR) state (MAR index = 1.00). To address this, our novel real-time SYBR Green PCR configuration successfully captures the specific thermodynamic signatures of these plasmid-borne integrons within hours of sampling, bypassing slow culture-based setups.

Nonetheless, several limitations must be acknowledged when interpreting these findings. Because this study relied on an exploratory statistical design with a modest sample size collected over a localized geographic area within a single season, these trends may not fully represent broader national epidemiology. Chronologically, while prospective sample collection was integrated into routine diagnostic workflows, experimental laboratory analysis of the clinical strains was executed entirely after receiving formal clearance from the institutional Research Ethics Committee. Additionally, while the critical polymyxin class was definitively validated using reference broth microdilution Minimum Inhibitory Concentrations (MICs), susceptibility testing for the remaining drug categories relied on disk diffusion screening. In the absence of high-resolution Whole Genome Sequencing (WGS), plasmid sequencing, or functional genetic assays, the underlying molecular configurations and transmission routes remain hypothesized. Future long-read genomic surveillance on expanded, multi-regional cohorts is highly recommended to fully map these plasmidic backbones. Implementing our proactive molecular framework across livestock, retail food, and clinical channels remains a vital component in tracking and disrupting the mobile networks driving the global emergence of untreatable pathogens.

## Figures and Tables

**Table 1:** Target Genes, De Novo Designed Primer Sequences, Accession Numbers, and Amplicon Sizes for the Optimized Real-Time qPCR Assays (T_anneal_ = 60 °C).

Target Gene	Primer Name	Primers Sequences (5′–3′)	Amplicon Size (bp)	Gene Accession No.
*intI1*	*intI1*-F	F; GCTCCAGTGCATTGACCAAA	185	AB104852.1
	*intI1*-R	R; GAGGCCACGGTCAAACAATT		
*intI2*	*intI2*-F	F; GGACAGGTCGATGATGGGAT	151	AF071413.3
	*intI2*-R	R; AGCACCTCAAGACATCAGCT		

**Table 2:** Prevalence of *Pseudomonas aeruginosa* Isolated from Different Samples.

Sample Source	Sample Type	Total No. of *P. aeruginosa* (%) ^a^
Human (*n* = 61)		20 (32.8)
	Blood (*n* = 29)	10 (34.5)
	Burn wound (*n* = 11)	4 (36.4)
	Urine (*n* = 21)	6 (28.6)
Food (*n* = 55)		22 (40)
	Minced meat (*n* = 30)	12 (40)
	Raw milk (*n* = 25)	10 (40)
*p*-value ^b^		0.445
Total (*n* = 116)		42 (36.2)

^a^ The isolation rate was calculated concerning the total number of the examined samples from each sample source. ^b^ *p*-value among isolates from different sample sources.

**Table 3:** Antibiotic Resistance Patterns of *Pseudomonas aeruginosa* Isolates from Different Sources.

Antimicrobial Class	AMA	No. of Resistant *P. aeruginosa* Isolates (%)		*p*-Value	Total No. of Resistant *P. aeruginosa* isolates (%) (*n* = 42)
		Food (*n* = 22)	Human (*n* = 20)		
**Aminoglycosides**	AK	12 (54.5)	12 (60.0)	0.764	24 (57.1)
	CN	20 (90.9)	20 (100)	0.489	40 (95.2)
	TOB	22 (100)	20 (100)	NA	42 (100)
**Carbapenems**	IPM	14 (63.6)	12 (60.0)	1	26 (12.8)
	MEM	22 (100)	20 (100)	NA	42 (100)
**Cephalosprins**	CAZ	22 (100)	20 (100)	NA	42 (100)
	FEP	14 (63.6)	10 (50)	0.533	24 (57.1)
**Fluoroquinolones**	LEV	16 (72.7)	18 (90)	0.243	34 (81)
	CIP	14 (63.6)	16 (80)	0.315	30 (71.4)
**Penicillins/beta-lactamase inhibitors**	TZP	12 (54.5)	16 (80)	0.108	28 (66.7)
	TIM	18 (81.8)	14 (70)	0.477	32 (76.2)
**Monobactams**	ATM	22 (100)	20 (100)	NA	42 (100)
**Polymyxins**	PB	22 (100)	20 (100)	NA	42 (100)
	CT	16 (72.7)	18 (90)	0.243	34 (81)
**β-lactam/β-lactamase inhibitor combinations**	CZA	22 (100)	20 (100)	NA	42 (100)
	C/T	22 (100)	20 (100)	NA	42 (100)

AMA: antimicrobial agent; NA: non-applicable.

**Table 4:** Frequency of Resistance to Various Antimicrobial Agents in *Pseudomonas aeruginosa* Isolates Belonging to Various Sources.

MARI	Resistant AMA	No. of *P. aeruginosa* Isolates (%)		*p*-Value	Total No. of *P. aeruginosa* Isolates (%) (*n* = 42)	Resistance Category
		Food (*n* = 22)	Human (*n* = 20)			
0.63	10	2 (9.1)	0	0.489	2 (4.8)	XDR
0.69	11	4 (18.2)	0	0.109	4 (6.5)	
0.75	12	4 (18.2)	9 (45)	0.096	13 (30.9)	
0.81	13	2 (9.1)	1 (5)	1	3 (7.1)	
0.88	14	4 (18.2)	1 (5)	0.346	5 (11.9)	
0.94	15	0	3 (15)	0.099	3 (7.1)	
1	16	6 (27.3)	6 (30)	1	12 (28.6)	PDR

AMA: antimicrobial agent.

**Table 5:** Frequency of Resistance to Various Antimicrobial Classes in *Pseudomonas aeruginosa* Isolates Belonging to Various Sources.

Resistant AMC	No. of *P. aeruginosa* Isolates (%)		*p*-Value	Total No. of *P. aeruginosa* Isolates (%) (*n* = 42)
	Food (*n* = 22)	Human (*n* = 20)		
7	4 (18.2)	2 (10)	0.665	6 (14.3)
8	18 (81.8)	18 (90)	0.665	36 (85.7)

AMC: antimicrobial class.

## Data Availability

The datasets supporting the findings of this study are available within the article and its Supplementary Materials. Correspondence and requests for further materials should be addressed to A.S.E-D.

## Supplementary Materials

The following supporting information can be downloaded at: https://www.mdpi.com/article/10.3390/pathogens15080788/s1, Supplementary Table S1. Minimum Inhibitory Concentration (MIC) distributions for Polymyxin B and Colistin against resistant *Pseudomonas aeruginosa* isolates determined via reference broth microdilution. Supplementary Table S2. Occurrence of various resistance categories in *Pseudomonas aeruginosa* isolates from different samples. Supplementary Table S3. Frequency of integron genes in *Pseudomonas aeruginosa* isolates from different sources. Supplementary Table S4. Distribution of the Class 2 integron gene (*int2*) gene between resistance phenotypes (XDR and PDR). Figure S1. Correlation plot showing the pairwise correlation (ρ) between phenotypic antimicrobial resistance, resistance categories, and integron of *Pseudomonas aeruginosa* isolates from different sources. The scale on the right of the figure refers to the correlation coefficient (ρ). The more intense the color, the more the stronger the positive or negative correlation.
