# Supplementary material for: A Novel Real-Time SYBR Green PCR Assay for Integron Profiling in Pseudomonas aeruginosa: Unveiling the Resistance Nexus Between Food and Clinical Isolates
Source: Pathogens. 2026 Jul 24;15(8):788. doi: 10.3390/pathogens15080788 (PMC13516557; doi:10.3390/pathogens15080788)
Supplement: Supplementary file 1 [file pathogens-15-00788-s001.zip › pathogens-4435657-supplementary.pdf]

Supplementary Table S1. Minimum Inhibitory Concentration (MIC) distributions for Polymyxin B and Colistin against resistant *Pseudomonas aeruginosa* isolates determined via reference broth microdilution.

Antimicrobial Agent	Resistant Isolates Tested (n)	MIC Range (µg/mL)	MIC50 (µg/mL)	MIC90 (µg/mL)	CLSI Breakpoint (≥µg/mL)	Phenotypic Interpretation
Polymyxin B (PB)	42	64 to ≥128	64	≥128	≥4	100% Confirmed Resistant
Colistin (CT)	34	32 to ≥128	64	≥128	≥4	100% Confirmed Resistant

Supplementary Table S2. Occurrence of various resistance categories in *Pseudomonas aeruginosa* isolates from different samples.

Resistant category	No. of *P. aeruginosa* isolates (%)		*p*-value	Total no. of *P. aeruginosa* isolates (%) (n= 42)
	Food (n= 22)	Human (n= 20)		
XDR	16 (72.7)	14 (70)	1	30 (71.4)
PDR	6 (27.3)	6 (30)	1	12 (28.6)

Supplementary Table S3. Frequency of integron genes in *Pseudomonas aeruginosa* isolates from different sources.

Criteria	gene	No. of *P. aeruginosa* isolates (%)		*p*-value	Total no. of *P. aeruginosa* isolates (%) (n= 42)
		Food (n= 22)	Human (n= 20)		
Class 1 integron gene	*int1*	22 (100)	20 (100)	NA	42 (100)
Class2 integron gene	*Int2*	12 (54.5)	14 (70)	0.354	26 (61.9)

NA: non-applicaple.

Supplementary Table S4. Distribution of the Class 2 integron gene (*intI2*) gene between resistance phenotypes (XDR and PDR).

Resistance category * int2 Crosstabulation

Count

		int2		
		Negative	Positive	Total
Resistance category	PDR	0	12	12
	XDR	16	14	30
Total		16	26	42

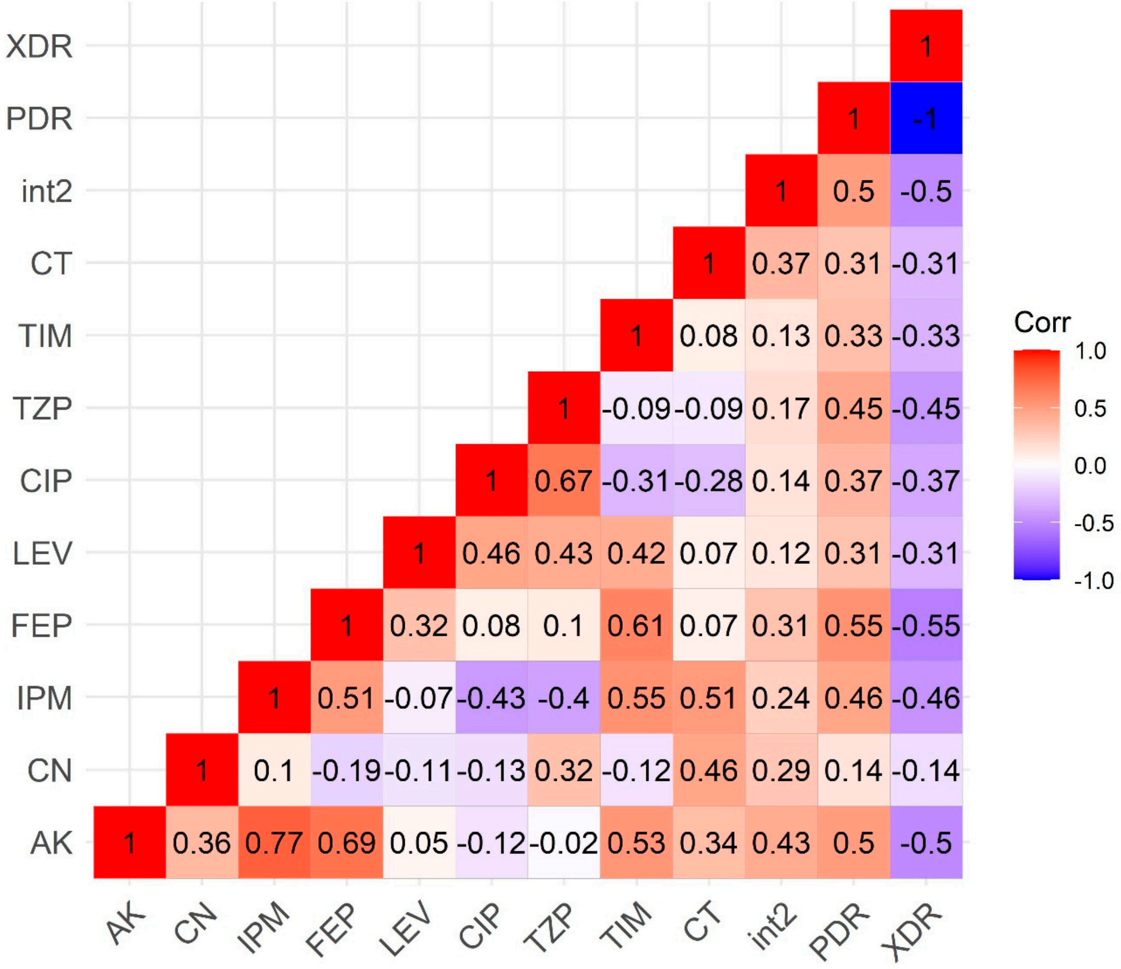

Figure S1. Correlation plot showing the pairwise correlation (ρ) between phenotypic antimicrobial resistance, resistance categories, and integron of *Pseudomonas aeruginosa* isolates from different sources. The scale on the right of the figure refers to the correlation coefficient (ρ). The more intense the color, the more the stronger the positive or negative correlation.
